# Changes in Body Weight, Body Composition, Phase Angle, and Resting Metabolic Rate in Male Patients with Stage IV Non-Small-Cell Lung Cancer Undergoing Therapy

**DOI:** 10.3390/medicina58121779

**Published:** 2022-12-02

**Authors:** Paraskevi Detopoulou, Theodora Tsiouda, Maria Pilikidou, Foteini Palyvou, Eirini Tsekitsidi, Maria Mantzorou, Persefoni Pezirkianidou, Krystallia Kyrka, Spyridon Methenitis, Gavriela Voulgaridou, Pavlos Zarogoulidis, Rena Oikonomidou, Dimitris Matthaios, Κonstantinos Porpodis, Dimitrios Giannakidis, Sousana K. Papadopoulou

**Affiliations:** 1 Department of Clinical Nutrition, General Hospital Korgialenio Benakio, 57889 Athens, Greece; 2 Department of Nutritional Sciences and Dietetics, University of the Peloponnese, 24150 Kalamata, Greece; 3 Pulmonary-Oncology Department, ‘Theageneio’ Cancer Hospital, 54639 Thessaloniki, Greece; 4 Department of Nutritional Sciences and Dietetics, International Hellenic University, 57001 Thessaloniki, Greece; 5 Department of Food Science and Nutrition, University of Aegean, 81100 Lemnos, Greece; 6 Sports Performance Laboratory, School of Physical Education and Sports Science, National and Kapodistrian University of Athens, 57668 Athens, Greece; 7 Pulmonary Department, General Clinic Euromedica Private Hospital, 54645 Thessaloniki, Greece; 8 3rd Surgery Department, AHEPA University Hospital, Aristotle University of Thessaloniki, 54637 Thessaloniki, Greece; 9 Health Center of Evosmos, 56224 Thessaloniki, Greece; 10 Oncology Department, General Hospital of Rhodes, 86775 Rhodes, Greece; 11 Pulmonary Department, “G. Papanikolaou” General Hospital, Aristotle University of Thessaloniki, 57010 Thessaloniki, Greece; 12 1st Department of Surgery, Attica General Hospital “Sismanogleio–Amalia Fleming”, 15126 Athens, Greece

**Keywords:** lung cancer, diet, phase angle, body composition, chemotherapy, radiotherapy, immunotherapy, Mediterranean diet

## Abstract

*Background and Objectives*: Cancer treatments can adversely influence body weight status, body composition, phase angle (PhA), and resting metabolic rate (RMR), which could possibly affect disease course. Τhe aim was to assess differences in body composition, PhA, RMR, and related parameters in non-small-cell lung cancer (NSCLC) patients after treatment. *Methods*: The sample consisted of 82 NSCLC (stage IV) male patients (chemotherapy (C) 15.7%; immunotherapy (I) 13.3%; C + I 25.3%; (C) + radiotherapy (R) 22.9 %; and other 15.5%). Body weight and body composition, PhA, RMR, oxygen consumption (VO_2_), ventilation rate, and diet were assessed at baseline and at 3 months after initiation of therapy. *Results*: Reductions in PhA, RMR, VO_2_, ventilation rate, and intracellular water were observed at follow up. Weight loss was evident for 45% of patients who also had a reduction in lean body mass. In the group under C, lean mass was reduced at follow up (55.3 ± 11.53 vs. 52.4 ± 12.6, *p* = 0.04) without significant weight changes. In subjects with a low adherence to the Mediterranean diet (MedDietScore < 30), RMR (1940 ± 485 vs. 1730 ± 338 Kcal, *p* = 0.001), VO_2_ (277.1 ± 70.2 vs. 247 ± 49.1 mL/min, *p* = 0.001), and ventilation rate (10.1 ± 2.28 vs. 9. ± 2 2.2 L/min, *p* = 0.03) were significantly reduced. The changes in body weight were positively related to % of change in fat mass (rho = 0.322, *p* = 0.003) and absolute lean mass change (rho = 0.534, *p* < 0.001) and negatively associated with % of change in total body water (rho = −0.314, *p* = 0.004) (Spearman correlation coefficients). *Conclusions*: In conclusion, cancer therapy related to reductions in PhA and RMR, while lean mass reduction may be related to the type of treatment. Our results emphasize the importance of a more holistic nutritional and body composition assessment beyond body weight, to better address patients’ needs in clinical practice.

## 1. Introduction

Lung cancer is the most common cause of cancer death with non-small-cell lung cancer (NSCLC) patients representing about 85% of new cases [1]. Cancer treatment can adversely influence body composition, causing metabolic derangements which affect disease course, [2,3] while potential adverse effects depend on the type of treatment [4]. Changes in several “simple” parameters, such as weight, body composition, and resting metabolic rate can have a prognostic value.

More particularly, body composition assessment with easy techniques such as bioelectrical impedance analysis (BIA) and the determination of indices, such as fat mass loss [5] and the phase angle (PhA) [6], can have a prognostic value in lung cancer, as recently reviewed by our team. Overweight [7] and weight gain [8,9,10,11] have been associated with reduced mortality in lung cancer patients, while body composition can affect tumor behavior, response to therapy, and therapy toxicity [12,13,14]. For example, radiotherapy may induce esophagitis, dysphagia, anorexia, and fatigue, and chemotherapy may induce gastrointestinal disturbances, all of which may affect nutrient intake and weight status [15,16]. 

Cancer type and staging, metabolic alterations, inflammation, and potential malnutrition and cachexia can also affect patients’ resting metabolic rate (RMR) [17]. Moreover, RMR and oxygen consumption (VO_2_) are reduced in lung cancer survivors, possibly because of depletion of fat and muscle mass [18] while evidence suggests high variability in RMR changes [19]. A possible related scenario suggests that RMR may initially increase but as catabolic pathways are activated RMR consequently declines [20]. This implies that RMR reflects disease severity and prognosis, as recently suggested by our group [18].

In this context, quantification of changes in body weight, body composition, phase angle, and RMR could have a prognostic capacity and is clinically important. Therefore, the aim of the present study was to assess the changes in body composition parameters and related variables in a sample of NSCLC patients before and after treatment and to detect possible differentiations across weight change patterns, treatments, and baseline dietary habits.

## 2. Methods

### 2.1. Study Design

This is a prospective study in which patients were assessed at baseline and after a 3-month follow up. All patients received first line treatment. The study received approval by the hospital’s investigational review board (protocol code 9817/12.6.2018) and all patients gave their written informed consent. The study was in accordance with the Declaration of Helsinki of 1975 (revised in 1983). More information on the study protocol can be found elsewhere [18]. Changes in body composition variables and RMR were tested in the whole sample. Moreover, stratifications were made according to weight changes, Mediterranean diet adherence and type of treatment, and changes in body composition and RMR were assessed in each stratum.

### 2.2. Patients

Eighty-two (*n* = 82) NSCLC stage IV patients from the Hospital ‘Theageneio’ (Thessaloniki, Greece) were assessed. All patients were male. All newly diagnosed patients were included at the time of study initiation. Included criteria: patients had to be ≥18 years of age and newly diagnosed with non-small-cell lung cancer (NSCLC) with histology type adenocarcinoma or squamous cell carcinoma with ECOG 0-2 biologically able to have any kind of treatment. Exclusion criteria were patients with any other histology type such as carcinoma, small-cell lung cancer (SCLC) and non-other specific (NOS). The percentage of every histologic subtype in this NSCLC population was 35 adenocarcinoma and 14 squamous cell carcinoma. Based on our department experience, we administered as chemotherapy carboplatin AUC 5.5 and paclitaxel 175 mg/m(2)/wk. Regarding immunotherapy, we administered nivolumab 3 mg/kg/2 weeks or pembrolizumab 2 mg/kg/3 weeks dosage. Regarding targeted treatment, we provided tyrosine kinase inhibitors for epidermal growth factor receptor positive patients. 

### 2.3. Anthropometric Measurements

Weight was measured with a digital scale (SECA 769, Hamburg, Germany) and height with a stadiometer (SECA 220, Hamburg, Germany). Measurements were taken in light clothing and without shoes. Body mass index (BMI) was then calculated as the ratio of weight (kg) divided by height squared (m^2^). Waist circumference (cm) was measured after a moderate expiration between the superior iliac crest and the lower rib margin in the midaxillary line. Hip circumference (cm) was measured at the level of the buttocks as the maximal horizontal circumference. Waist to hip ratio was then determined. 

### 2.4. Body Composition Measurements

Body composition was assessed with the BIA method by using Bodystat Quadscan 4000 (Ballakaap, UK) which is a tetrapolar and multiple-frequency equipment measuring impedance at 5 kHz, 50 kHz, 100 kHz, and 200 kHz. The measurement was performed according to the instructions of the manufacturer by attaching two sensing electrodes to the wrist and ankle and two current electrodes to the dorsum of hand and foot (right side of patient). Percentage of total body fat (BF%), total lean mass (kg), total body water (%), extracellular water (%), and intracellular water (%) were estimated by sex-specific equations built into the equipment. Phase angle (at 50 Khz) was calculated as follows:

Phase angle = (resistance/reactance) × (180/π).

### 2.5. Resting Metabolic Rate (RMR) Measurement and Related Parameters

RMR measurement was done with the portable indirect calorimeter Fitmate GS (Cosmed, Rome, Italy). Subjects were asked to lie in a supine position and rest for 20 min in a silent room. Calibration was performed before each measurement. VO_2_ and ventilation rate were also assessed in mL/min and L/min, respectively.

### 2.6. Lifestlyle Variables

A short 11-item food frequency questionnaire (FFQ) was filled in and the Mediterranean Diet Score (MedDietScore) was calculated [21]. The score ranges from 0 to 55 with greater numbers indicating higher adherence to the Mediterranean diet. For the assessment of physical activity, patients filled in the International Physical Activity Questionnaire (IPAQ) questionnaire (short form) [22]. Moreover, smoking habits were assessed. The number of cigarettes and the years of smoking were recorded. Then, pack years were assessed as a measure of tobacco exposure for each subject by multiplying the number of packs of cigarettes per day with smoking years.

### 2.7. Statistical Analysis

Normality was tested with the Kolmogorov–Smirnoff criterion. Normally distributed continuous variables are presented as mean values ± standard deviation (SD), while skewed variables were presented as median and interquartile range. Categorical variables are presented as relative frequencies (%). 

All reported *p*-values were two-sided (significance level 5%). SPSS v22 software was used for statistical analysis (IBM Corp. Released 2013, IBM Corp., Armonk, NY, USA). Variables are presented as median and interquartile range (25th–75th). Binary variables are presented as percentages. Changes between baseline and follow-up values were tested with the paired *t* test (for normal variables) of the Wilcoxon non-parametric test (for non-normal variables). Spearman correlation coefficient was chosen to assess correlations between changes in variables since some data were skewed and correlations may be non-linear. The level of statistical significance was set at 5%. The Statistical Package for the Social Sciences (SPSS 18.0 for Windows, Chicago, IL, USA) was used for all the analyses. Post hoc power analysis was performed with the statistical software G*power (version 3.1.9.7, Universität Kiel, Kiel, Germany).

## 3. Results

### 3.1. Baseline Characteristics

The characteristics of patients with lung cancer are shown in Table 1. It is noted that only two subjects had moderate physical activity and the rest of them had low physical activity. The mean BMI ± SD of subjects was 26.9 ± 5 kg/m^2^. The mean ± SD for total lean mass was 57.4 ±10.6 kg and for PhA was 5.1 ± 0.8°. Subjects were under various treatments.

### 3.2. Changes in Weight, Body Composition, and RMR Parameters in the Whole Sample

In Table 2, the changes in weight, body composition, RMR, and related parameters are displayed for the study population. Weight loss was observed in 37 patients (45%) and weight gain was observed in 17 patients (20.7%), while stable weight was recorded in 27 patients (32.9%). It is noted that weight loss >2% was documented in 23 patients (27.7%). PhA, intracellular water, RMR, VO_2_, and ventilation rate were significantly reduced at follow up.

### 3.3. Changes in Weight, Body Composition, and RMR Parameters according to Weight Change Stratification

**Figure 1:**
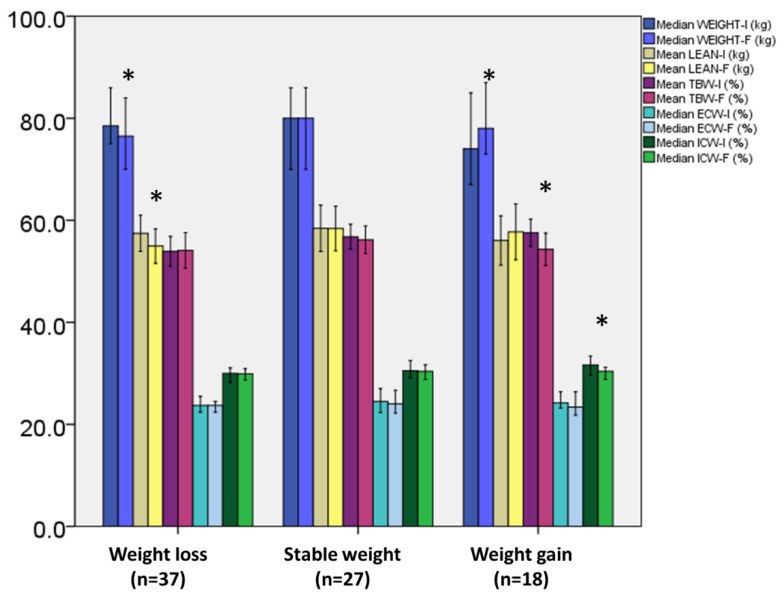
Changes in body weight, lean mass and hydration status according to weight changes. Bars represent mean or median values and 95% confidence intervals. * denotes *p*-value< 0.05. I: initial; F: final; TBW: total body water; ECW: extracellular water; and ICW: intracellular water.

In Figure 1, changes in body weight, lean mass, and body water status are shown across several categories of weight changes, i.e., weight loss, weight stability, and weight gain. As is shown, weight loss was connected to loss of lean mass. Patients who gained weight experienced decreases in % of total body water and intracellular water. In Table 3, the changes in % of body fat, phase angle, resting metabolic rate, and related parameters are displayed for several categories of weight changes. In the groups with stable weight or weight gain, decreases in RMR, VO_2_, and ventilation rate were noted. Moreover, in all groups, irrespective of weight changes, PhA was lower at the follow up. 

### 3.4. Changes in Weight, Body Composition, and RMR Parameters according Baseline Diet

In subjects with a low adherence to the Mediterranean diet (1st tertile of the MedDietScore with scores < 30), it was observed that RMR (1940 ± 485 vs. 1730 ± 338 Kcal, *p* = 0.001), VO_2_ (277.1 ± 70.2 vs. 247 ± 49.1 mL/min, *p* = 0.001), and ventilation rate (10.1 ± 2.28 vs. 9.2 ± 22.2 L/min, *p* = 0.03) were significantly reduced, while no changes were observed in patients on the other tertiles (data not shown).

### 3.5. Changes in Weight, Body Composition, and RMR Parameters according to Medical Treatment

Furthermore, changes in body composition and RMR were studied according to most common treatment routes in our sample, i.e., chemotherapy, immunotherapy, chemotherapy + immunotherapy, and chemotherapy + radiotherapy in a subgroup of 64 patients. In the subgroup of chemotherapy, lean mass was reduced at follow up (55.3 ± 11.53 vs. 52.4 ± 12.6 kg, *p* = 0.04) without significant weight changes. In the group of chemotherapy + immunotherapy, there was a trend for weight reduction (78.9 ± 15.6 vs. 77.1 ± 15.7 kg, *p* = 0.06) and a significant reduction in RMR (2068 ± 620 vs. 1740 ± 487 Kcal, *p* = 0.03). In all groups, PhA was lower at the follow up. The rest of the investigated variables did not change between the baseline and follow up in this subgroup analysis (data not shown).

### 3.6. Correlations between Changes in Body Composition, PhA, and MedDietScore

**Figure 2:**
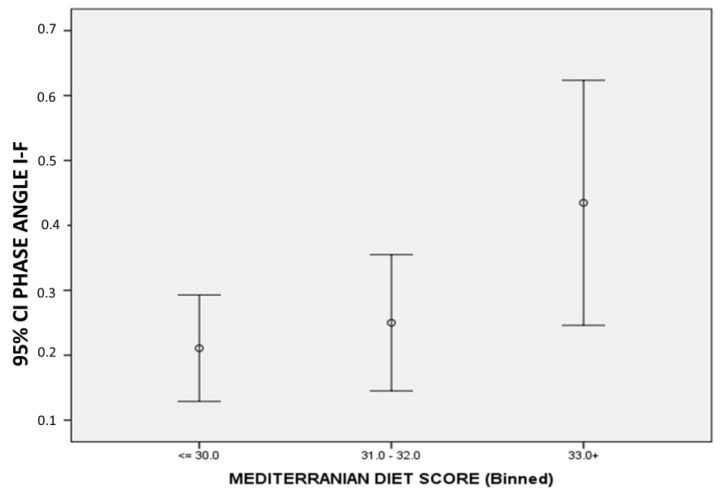
Relation of baseline MedDietScore to changes in phase angle during therapy. Changes in phase angle represent differences between initial and final values. MedDietScore: Mediterranean diet score.

In Table 4, Spearman correlation coefficients of changes in the investigated variables are shown. Body weight changes were positively related to both fat mass and lean mass changes and negatively associated with total body water and ECW. MedDietScore was positively related to phase angle changes (initial–final), which is also illustrated in Figure 2.

## 4. Discussion

The present study describes the changes in body weight, body composition parameters, PhA, and RMR in stage IV NSCLC male patients after cancer treatment and investigates the possible role of type of treatment and baseline dietary habits. Variable responses were documented for weight and body weight changes that were positively related to lean mass and fat changes. PhA and RMR were reduced after cancer treatment. Low Mediterranean diet adherence at baseline related to differences in PhA and reductions in RMR. In addition, chemotherapy was related to reductions in lean mass without reductions in body weight, while in chemotherapy- and immunotherapy-treated patients, RMR was reduced without a reduction in lean mass.

The assessment of weight changes is important for prognosis in lung cancer patients since weight gain [8,9,10,11] has been associated with reduced mortality in lung cancer patients and patients with advanced NSCLC, in particular [23]. In our study variable responses to weight were observed, which is in line with previous studies [24]. From a clinical perspective, a weight loss >2% has been shown to predict mortality in cancer patients [25] and in our study, almost 30% of patients experienced such weight loss. In other studies, 30–34.5% of patients had severe weight loss (>5–10% of body weight), which is comparable to our results [26] or were in high nutritional risk [27]. Weight changes in the whole sample were related to lean and fat mass alterations, as expected. is particularly important for clinical practice in which usually only body weight measurements However, in the treatment subgroup analysis, it was found that in the chemotherapy subgroup lean mass was reduced without changes in body weight. This observation are recorded, and thus lean mass perturbations may be masked. This means that apart from weight, lean mass should be also assessed. Furthermore, muscle mass reservoirs may be related to a decrease in infections in hospitalized patients [28] and better distribution of chemotherapy implying lower toxicity [29].

Another parameter relating to body composition that deserves special attention is PhA. PhA has been positively associated with functional fitness and better physical function in older adults [30], which depends on muscle mass [31] and has also been related to lung cancer prognosis [6]. PhA was reduced after cancer treatment irrespective of the line of treatment. A potential explanation for the observed reduction in PhA is the reduction in muscle mass, while it is also possible that the reduction in PhA goes along with disease progression. A higher MedDietScore was related to larger differences (PhA initial–PhA final) thus indicating lower final PhA values, which is in agreement with findings that the Mediterranean diet is related to higher PhA values in healthy subjects [32]. It is also noted that our group recently revealed a relation to diet with PhA in lung cancer patients [3]. 

The interpretation of RMR changes in cancer patients is quite challenging [17]. On the one hand, weight and lean mass changes alter basal metabolic needs [33], and on the other hand, inflammation may increase energy expenditure in advanced-stage patients [34]. On top of these, lung disease and possibly diagnosed or undiagnosed chronic obstructive pulmonary disease may reduce VO_2_ consumption and related measured energy expenditure. In the present study, RMR was reduced even for patients who experienced weight stability or gain, which has been observed in cancer patients [35]. This has implications for clinical practice since energy requirements are usually calculated with equations with the use of several variables including weight [17]. Moreover, according to our results, a lower adherence to the Mediterranean diet was connected to reductions in RMR, implying a possible increased inflammatory state [17]. Indeed, in NSCLC patients, inflammatory markers were negatively associated with circulating antioxidants, such as retinol, alpha-tocopherol, and lutein [36]. Of course, the total energy needs assessment requires more attention since it is affected by physical activity status. Indeed, total energy needs could be even lower since tumor-related phenotypes, such as fatigue, may further decrease energy expenditure [37].

The strengths of our study include the homogeneity of the investigated population (males, stage IV NSCLC patients) and the measurement of a panel of body composition variables as well as RMR along with the assessment of dietary habits. RMR was not measured with indirect calorimetry but the used Fitmate GS calorimeter performs well at group level in cancer patients [38]. Post hoc power analysis showed that the sample size is adequate to evaluate two-sided standardized differences between the observed changes in resting metabolic rate, achieving statistical power greater than 0.81 at 5% probability level (*p*-value). Power analysis was performed with the statistical software G*power (version 3.1.9.7, Universität Kiel).

However, several limitations should be considered. It was a single-center study, which may imply some selection bias. Data on the nutritional support of patients were not available which may affect weight loss, and related measurements and biochemical indices were not recorded. In addition, we have no data on segmental PhA measurements. However, it has been shown that whole body PhA is a better predictor of malnutrition in cancer patients when several measurements are compared [39]. Last but not least, possible metastasis data were not available in the present analysis.

In conclusion, cancer therapy is related to a reduction in PhA, RMR, and related parameters. RMR reductions may depend on baseline diet and take place irrespective of weight changes. Lean mass reduction may be related to the type of treatment. Our results emphasize the importance of a more holistic nutritional and body composition assessment beyond body weight, to better address patients’ needs in clinical practice.

## Figures and Tables

**Table 1:** Basic characteristics of participants and lines of treatment.

	Total (*n* = 82)	
	Mean or Median	SD or 25th–75th
Age (years)	65.8	9.1
Pack years	75.5	47.5–102.5
BMI (kg/m^2^)	26.9	5.0
Waist circumference (cm)	105.0	96.0–120.0
Hip circumference (cm)	104.0	98.0–111.2
Waist-to-hip ratio	1.04	0.94–1.10
MedDietScore	31.0	29.0–33.0
	**Frequency (n)**	**%**
Chemotherapy (C)	13	15.7
Immunotherapy (I)	11	13.3
C + I	21	25.3
C +targeted treatment *	5	6.0
C+ Radiotherapy (R)	19	22.9
I + R	4	4.8
R + P + I + P	3	3.6
C + R + P	1	1.2

Data are presented as mean ± standard deviation for normally distributed variables. Otherwise, data are presented as median (lower–upper quartile) (25th–75th). Chemotherapy (C), immunotherapy (I), and radiotherapy (R). * represents targeted treatment. Based on our department experience, we administered as chemotherapy carboplatin AUC 5.5 and paclitaxel 175 mg/m(2)/wk. Regarding immunotherapy, we administered nivolumab 3mg/kg/2weeks or pembrolizumab 2 mg/kg/3 weeks dosage. Regarding targeted treatment, we provided tyrosine kinase inhibitors for epidermal growth factor receptor positive patients such as erlotinib and gefitinib.

**Table 2:** Changes in body composition and resting metabolic rate after treatment in all patients(*n* = 82).

	Baseline	Follow Up	
	Mean ± SD or Median, 25th–75th	Mean ± SD or Median, 25th–75th	*p*
Weight (Kg)	78.0, 70–87.2	77.0, 68.0–87.0	0.07
BMI (kg/m^2^)	26.9 ± 5.0	26.9± 5.7	0.9
Waist circumference (cm)	105.0, 96.0–120.0	107.0, 97.0–120.0	0.7
Total body fat (%)	27.8 ± 7.1	28.5 ± 7.3	0.2
Total lean mass (kg)	57.4 ± 10.6	56.6 ± 10.60	0.1
Total body water (%)	55.6 ± 7.4	54.8 ± 8.5	0.4
Extracellular water (%)	24.2, 22.1–26.6	23.8, 21.8–26.4	0.2
Intracellular water (%)	30.4, 29.0–32.7	30.1, 28.4–31.6	0.009
PhA (^o^)	5.1 ± 0.8	4.9 ± 0.8	<0.0001
Resting metabolic rate (Kcal)	1869 ± 414	1743 ± 367	0.002
VO_2_ (mL/min)	267.7 ± 60.7	248.3 ± 53.3	0.002
Ventilation rate (L/min)	9.98 ± 2.08	9.41 ± 2.28	0.03
Respiratory frequency (breaths/min)	19.35 ± 4.78	19.50 ± 4.03	0.7
Fraction of exhaled oxygen (%)	17.7 ± 0.44	17.7 ± 0.54	0.8

Data are presented as mean ± standard deviation for normally distributed variables. Otherwise, data are presented as median (lower–upper quartile) (25th–75th). BMI: body mass index; PhA: phase angle; and VO_2_: oxygen consumption.

**Table 3:** Changes in body composition and resting metabolic rate according to weight changes.

	Baseline	Follow Up	*p*
	Mean ± SD or Median, 25th–75th	Mean ± SD or Median, 25th–75th	
*Subjects with weight loss (n = 37)*			
BMI (kg/m^2^)	27.4 ± 5.4	26.1 ± 5.2	<0.0001
Total body fat (%)	29.38 ± 9.04	29.37 ± 6.01	0.9
Waist circumference (cm)	101.0, 95.5–118.5	106.0, 97.0–119.0	0.8
Hips circumference (cm)	103.0, 98.0–113.0	102.0, 97.5–109.0	0.06
PhA (^o^)	5.15 ± 0.75	4.85 ± 0.73	<0.0001
Resting metabolic rate (Kcal)	1862 ± 419	1797 ± 375	0.2
VO_2_ (mL/min)	265.5 ± 62.9	255.9± 54.8	0.2
Ventilation rate (L/min)	9.79 ± 2.14	9.81 ± 2.31	0.9
Respiratory frequency (breaths/min)	18.81 ± 5.64	19.91 ± 4.61	0.1
Fraction of exhaled oxygen (%)	17.74 ± 0.50	17.7 ± 0.57	0.4
*Subjects with stable weight (n = 27)*			
BMI (kg/m^2^)	27.5 ± 5.5	27.1 ± 5.5	1.0
Total body fat (%)	26.62 ± 8.32	26.7 ± 8.85	0.6
Waist circumference (cm)	110.0, 96.0–122.0	107.0, 94.0–122.0	0.3
Hips circumference (cm)	106.0, 98.0–113.0	105.0, 93.0–110.0	0.3
PhA (^o^)	5.16 ± 0.90	4.93 ± 0.89	<0.0001
Resting metabolic rate (Kcal)	1767 ± 385	1650 ± 351	0.05
VO_2_ (mL/min)	254.2 ± 55.2	234.8 ± 50.8	0.04
Ventilation rate (L/min)	9.6 ± 2.0	8.9 ± 2.2	0.03
Respiratory frequency (breaths/min)	19.0 ± 3.8	18.9 ± 3.7	0.8
Fraction of exhaled oxygen (%)	17.8 ± 0.46	17.8 ± 0.60	0.8
*Subjects with weight gain (n = 18)*			
BMI (kg/m^2^)	25.7, 24.1–27.2	25.3, 26.9–29.2	<0.0001
Total body fat (%)	26.68 ± 4.92	29.61 ± 7.41	0.08
Waist circumference (cm)	107.0, 100.0–115.7	108.0, 101.0–114.5	0.2
Hips circumference (cm)	101.5, 98.2–110.2	105.5, 101.2–110.0	0.3
PhA (^o^)	5.30 ± 0.86	4.96 ± 0.85	0.03
Resting metabolic rate (Kcal)	2035 ± 415	1771 ± 365	0.03
VO_2_ (mL/min)	292.2 ± 59.80	253.2 ± 53.0	0.02
Ventilation rate (L/min)	10.8 ± 1.91	9.2 ± 2.1	0.04
Respiratory frequency (breaths/min)	20.8 ± 3.9	19.4 ± 3.08	0.1
Fraction of exhaled oxygen (%)	17.7 ± 0.26	17.6 ± 0.39	0.4

Changes between baseline and follow-up values were tested with the paired *t* test (for normal variables) of the Wilcoxon non-parametric test (for non-normal variables). Data are presented as mean ± standard deviation or median, lower-upper quartile (25th–75th). BMI: Body mass index; PhA: phase angle; VO_2_: oxygen consumption.

**Table 4:** Spearman correlation coefficients between changes in weight, body composition variables, and MedDietScore.

		Weight Change (kg)	BMI Change (kg/m²)	Waist Circumference Change (cm)	Fat Change (%)	Lean Mass Change (kg)	TBW Change (%)	ECW Change (%)	Phase Angle Change (°)
Weight change (kg)	rho		0.998	0.193	0.322	0.534	−0.314	−0.252	−0.01
	p		<0.001	0.08	0.003	<0.001	0.004	0.02	0.9
BMI change (kg/m²)	rho	0.998		0.199	0.328	0.527	−0.320	−0.260	−0.02
	p	<0.001		0.07	0.003	<0.001	0.003	0.01	0.8
Waist circumference change (cm)	rho	0.193	0.199		0.268	−0.01	−0.14	−0.03	−0.08
	p	0.08	0.07		0.01	0.9	0.2	0.7	0.4
Total body fat change (%)	rho	0.322	0.328	0.268		−0.359	−0.749	−0.615	−0.12
	p	0.003	0.003	0.01		<0.001	<0.001	<0.001	0.2
Lean mass change (kg)	rho	0.534	0.527	−0.01	−0.359		0.202	0.147	0.201
	p	<0.001	<0.001	0.9	<0.001		0.06	0.1	0.07
TBW change (%)	rho	−0.314	−0.320	−0.14	−0.749	0.202		0.831	0.137
	p	0.004	0.003	0.2	<0.001	0.06		<0.001	0.2
ECW change (%)	rho	−0.252	−0.260	−0.03	−0.615	0.147	0.831		0.116
	p	0.02	0.01	0.7	<0.001	0.1	<0.001		0.2
PhA (^o^)	rho	−0.01	−0.02	−0.08	−0.117	0.201	0.137	0.116	
	p	0.9	0.8	0.4	0.2	0.07	0.22	0.2	
Mediterranean diet score	rho	−0.12	−0.13	−0.04	−0.154	−0.01	0.093	0.171	0.251
	p	0.2	0.2	0.7	0.1	0.9	0.4	0.1	0.02

Changes represent the difference in initial and final values (initial–final). Significant correlation coefficients are shown in bold. BMI: body mass index; PhA: phase angle; TBW: total body water; and ECW: extracellular water.

## Data Availability

The data are available if requested by the corresponding author.

## Abbreviations

BIA	B ioelectrical impedance analysis
MedDietScore	Mediterranean diet score
NSCLC	Non-small-cell lung cancer
PhA	Phase angle
R	Resistance
Xc	Capacitive reactance
